# Tuberculosis y hacinamiento carcelario desde la perspectiva de las inequidades sociales en salud en Colombia, 2018

**DOI:** 10.7705/biomedica.5894

**Published:** 2022-03-01

**Authors:** Martha Patricia López, Adriana Paola Ulloa, Fabio Alberto Escobar

**Affiliations:** 1 Maestría en Salud Pública y Desarrollo Social, Fundación Universitaria del Área Andina, Bogotá, D.C., Colombia Fundación Universitaria del Área Andina Fundación Universitaria del Área Andina Bogotá D.C Colombia

**Keywords:** tuberculosis, prisioneros, disparidades en el estado de salud, densidad de población, sistemas de salud, vigilancia en salud pública, Colombia, Tuberculosis, prisoners, disparities in health status, population density, health systems, public health surveillance, Colombia

## Abstract

**Introducción.:**

La población privada de la libertad se encuentra afectada por la tuberculosis debido al hacinamiento carcelario. Esta situación refleja una inequidad en salud, entendida esta como una diferencia injusta y evitable.

**Objetivo.:**

Estimar las condiciones de hacinamiento carcelario como inequidad en salud de la población privada de la libertad que padece tuberculosis en Colombia durante el 2018.

**Materiales y métodos.:**

Estudio ecológico para estimar las inequidades a nivel nacional en la población privada de la libertad, utilizando la guía metodológica de la Organización Mundial de la Salud (OMS) para la medición de desigualdades. A partir de los datos del sistema de vigilancia en salud pública, la incidencia de tuberculosis sirvió como indicador y el porcentaje de hacinamiento se usó para estratificar la equidad.

**Resultados.:**

La desigualdad relativa entre los quintiles de menor y mayor hacinamiento evidenció que la incidencia de la tuberculosis en la población privada de la libertad con mayor hacinamiento es 1,92 veces la del grupo con menor hacinamiento. El índice de desigualdad demostró un exceso de 724 casos de tuberculosis por cada 100.000 internos entre la población con mayor concentración de hacinamiento. El índice de concentración en salud fue de -0,121, lo que refleja que la incidencia se concentró en el grupo con más sobrepoblación.

**Conclusión.:**

En Colombia, la población privada de la libertad en condiciones de hacinamiento y que padece tuberculosis, enfrenta desigualdades injustas y evitables, comparada con quienes no están en esas condiciones. Se requieren políticas que reduzcan el hacinamiento y mejoren las condiciones de vida en las cárceles.

## Abstract (en)

**Introduction:**

Prison population is affected by tuberculosis (TB) due to prison overcrowding. This situation reflects an inequity in health, understood as an unfair and avoidable difference between individuals and populations.

**Objective:**

To estimate the conditions of prison overcrowding as health inequity in prison population suffering from TB in Colombia during 2018.

**Materials and methods:**

This is an ecological study to estimate inequities at the national level in prison population through the use of the World Health Organization methodology for the measurement of inequalities. Based on data from the public health surveillance system, the incidence of TB was taken as an indicator and the percentage of overcrowding as an equity stratifier. Simple and complex measures of inequality were calculated.

**Results:**

The relative inequality between the lowest and highest quintiles of crowding showed that TB incidence among prison population with the highest crowding was 1.92 times that of the group with the lowest crowding. The inequality index identified an excess of 724 TB cases per 100,000 among those with the highest concentration of overcrowding. The health concentration index was -0.121, which shows that the incidence was concentrated in the group with the most overpopulation.

**Conclusion:**

In Colombia, prison population in overcrowded conditions and suffering from TB has to face unfair and avoidable inequalities as compared with those not living in these conditions. Policies are required to reduce overcrowding and improve living conditions in prisons.

Las inequidades en salud hacen referencia a diferencias en ese ámbito que son evitables y socialmente injustas, entre individuos o grupos. Ahora bien, cualquier variación cuantificable puede considerarse una desigualdad en salud aunque se trate simplemente de una descripción que se emplea cada vez que las cantidades son diferentes ^1^. Estas desigualdades reflejan diferencias entre sociedades y grupos sociales en aspectos como la mortalidad, la enfermedad y el acceso a los servicios de salud, determinadas por las variaciones a nivel social o económico ^2^^-^^4^.

Según Margareth Whitehead, la inequidad en salud equivale al concepto de justicia relacionado con la situación de salud, la calidad de vida y la supervivencia individual. Idealmente, todas las personas deberían alcanzar su máximo potencial en salud y nadie debería estar en desventaja ^5^. El concepto responde a criterios relevantes para determinar la doble condición de evitabilidad y de injusticia inherente a la imposición de los riesgos, excepto en dos situaciones: exposición voluntaria (comportamientos de riesgo, deportes peligrosos, contingencias especiales) y riesgo estructural inevitable (edad, sexo, genética). La desigualdad comprende las principales diferencias dimensionales, mensurables, sistemáticas y evitables, entre los miembros de una población dada ^6^.

Teniendo en cuenta la perspectiva de las inequidades sociales en salud, la población privada de la libertad es un grupo poblacional que se encuentra en una situación de vulnerabilidad social que la expone a mayores riesgos en salud debido, entre otras razones, al hacinamiento o superpoblación carcelaria en las instituciones penitenciarias ^7^. Comparada con otros grupos poblacionales o la población en general, esta población enfrenta diferencias en sus condiciones de vida y de salud que son evitables e injustas, y constituyen inequidades sociales.

Cuando se abordan las condiciones de salud y de enfermedad de la población privada de la libertad, se suele subestimar la importancia de sus condiciones de vida particulares ^8^. En este sentido, en la década de 1990, la Organización Mundial de la Salud (OMS) y su oficina regional en Europa plantearon prestar especial atención a enfermedades como el VIH/sida y la tuberculosis en la población recluida en prisiones y otros sitios de detención a partir de una aproximación basada en la promoción de la salud orientada a la transformación de sus condiciones de vida ^9^.

Las condiciones de vida en prisión se han considerado como un factor social determinante en salud, dada la concentración, amplificación, deterioro, diseminación y sobrecarga de la morbilidad y la mortalidad ^10^. El hacinamiento carcelario incrementa el contacto físico, limita la ventilación y la iluminación, y dificulta el uso del tiempo en los espacios abiertos, factores que inciden en el riesgo de contraer enfermedades transmisibles como la tuberculosis. En estudios en Chile, Brasil y Sudáfrica, entre otros, se ha demostrado el efecto del hacinamiento en el aumento significativo de esta enfermedad en la población privada de la libertad comparada con la población general ^11^^-^^14^.

Según datos de la iniciativa “Alto a la tuberculosis”, cada año nueve millones de personas contraen la enfermedad y 1,8 millones de ellas fallecen, lo que la convierte en la principal causa de muerte por enfermedad infecciosa, a pesar de que se cuenta con un tratamiento gratuito y supervisado ^15^. Asimismo, el “Plan mundial para poner fin a la tuberculosis, 2018-2022”, formulado por la OMS, aspira a contribuir a la reducción de la carga de tuberculosis, incluido un enfoque específico para la población de prisiones y centros de detención, con el desarrollo de acciones basadas en un enfoque de derechos humanos que garantice que nadie sea excluido ^15^.

Además, la OMS estableció la estrategia “Fin a la tuberculosis, 2016-2035”, con base en tres pilares: la atención integral centrada en las personas, políticas audaces y sistemas de soporte e investigación e innovación operativa para avanzar hacia la eliminación de la enfermedad ^16^. En este sentido, Colombia adoptó políticas para el control y la eliminación de la enfermedad en el “Plan decenal de salud pública, 2012-2021” ^17^ y en el “Plan estratégico nacional colombia hacia el fin de la TB, 2016-2025” ^18^.

En dicho contexto, el objetivo del presente estudio fue estimar las condiciones de hacinamiento carcelario como factor de inequidad en salud en la población privada de la libertad que padece tuberculosis en Colombia durante el 2018.

## Materiales y métodos

Se llevó a cabo un estudio ecológico a partir de la información de casos de tuberculosis en todas sus formas (pulmonar, extrapulmonar, nuevos y previamente tratados) en población privada de la libertad notificados al Sistema de Vigilancia en Salud Pública (Sivigila) del Instituto Nacional de Salud mediante la ficha de datos básicos y complementarios bajo el código 813 durante las semanas epidemiológicas 01 a 52 del 2018. Las variables demográficas analizadas fueron edad, pertenencia etnia y régimen de salud; las variables clínicas incluyeron el tipo de tuberculosis y su clasificación según la historia clínica, y las variables sociales, el hacinamiento.

La metodología se basó en la guía de paso a paso para el cálculo de métricas de desigualdad en salud de la OMS: se partió de la selección de fuentes de información para catalogar los datos disponibles; se hizo el análisis combinando la información sobre los indicadores de salud y los estratificadores de equidad y el porcentaje de hacinamiento que se usó para estratificar la equidad, y se calculó el nivel promedio del indicador de salud para cada subgrupo. Teniendo en cuenta que se pueden utilizar diversas mediciones para analizar las desigualdades en salud, se tomó como indicador de salud la incidencia de tuberculosis y, para estratificar la equidad, el porcentaje de hacinamiento en cada centro penitenciario y carcelario del país.

Para el análisis de las desigualdades sociales, se calcularon las medidas simples de desigualdad como brechas absoluta y relativa, y diferencia y razón entre las tasas de incidencia de tuberculosis y el hacinamiento en cada centro penitenciario y carcelario, así como los índices de pendiente y de concentración, medidas complejas que permiten determinar la concentración de la carga de enfermedad en el extremo socialmente más desaventajado de la población y viceversa.

Se establecieron las medidas de frecuencias relativas y absolutas, los cuantiles y las tasas de incidencia por cada 100.000 personas. Para el análisis de tasas, se estableció como numerador el número de nuevos casos de tuberculosis a nivel nacional y local, en tanto que el denominador poblacional se construyó a partir del tamaño de la población privada de la libertad reportada por el Instituto Penitenciario y Carcelario (INPEC) para cada establecimiento a nivel local y nacional, con corte a diciembre del 2018. Se organizaron grupos por quintiles, teniendo en cuenta la tasa de incidencia de la enfermedad en la población privada de la libertad y el hacinamiento por centro carcelario, cuyo peso se determinó dentro de cada grupo (cuadro 1).

**Cuadro 1:** Centros carcelarios por quintiles

Quintil	Centro carcelario y penitenciario
Quintil 1	EPMSC Cartago
	EPMSC Acacías
	EPAMS Girón
	Complejo Carcelario y Penitenciario de Ibagué - Picaleña
	INPEC Líbano
	EPAMSCAS Popayán (ERE)
	EPMSC La Esperanza Guaduas
	Complejo Carcelario y Penitenciario de Jamundí
	EPAMS La Dorada
	EPMS San Gil
	EP Las Heliconias de Florencia
	EPMSC Tierralta
	EPMSC Bolívar-Cauca
	EPMSC Calarcá
	EPMSC Valledupar
Quintil 2	EPMSC Pitalito
	Complejo Carcelario y Penitenciario Metropolitano de Bogotá
	EPAMSCAS Cómbita
	EPMSC Anserma
	EPMSC Leticia
	EPMSC La Plata
	EP Puerto Triunfo
	EPMSC Santa Rosa de Cabal
	EPC Yopal
	EPMSC- Sevilla
	EPMSC Girardot
	EPMSC Armenia
	EPMSC Tuluá
	RM Pereira
	EPMSC Paz de Ariporo
Quintil 3	Complejo Penitenciario Medellín - Pedregal
	EPAMS Palmira
	Establecimiento Carcelario “La Modelo” - Puente Aranda
	EPMSC Florencia
	EPMSC Istmina
	Complejo Carcelario y Penitenciario Metropolitano de Cúcuta
	EPMSC Honda
	EPMSC Buga
	EPMS San Andrés
	EPMSC Garzón
	EPMSC Socorro
	Cárcel de Mujeres
	EPMSC Yarumal
	EPMSC Málaga
Quintil 4	EPMSC Santander de Quilichao
	EPMSC Sincelejo
	EPMSC Tumaco
	EPMSC Montería
	EPMSC Caucasia
	EPMSC Villavicencio
	EPMSC Puerto Berrío
	EPMSC Neiva
	EPMSC Pensilvania
	EPMSC Pereira (ERE)
	EPMSC Bucaramanga (ERE)
	RM Bucaramanga
	EPMSC Buenaventura
	EPMSC Cartagena
	EPMSC Medellín
Quintil 5	EPMSC Andes
	EPMSC Riohacha
	INPEC Cárcel Santa Marta
	EPAMSCAS Valledupar (ERM)
	EPMSC La Ceja
	EPMSC Cali (ERE)
	EPMSC Sonsón
	EPMSC Barranquilla
	EPMSC Barrancabermeja
	EPMSC Santo Domingo
	EPMSC RM Pasto
	EC Barranquilla
	EPMSC El Banco
	EPMSC Quibdó
	EPMSC Manizales

Para el análisis de la información, se empleó el programa estadístico SPSS^™^, versión 12.0 y, para la elaboración de tablas, se utilizó el programa Microsoft Excel^™^ 2003.

Según la Resolución 8430 de 1993 del Ministerio de Salud y Protección Social, la cual establece las normas académicas, técnicas y administrativas para la investigación en salud, el estudio se clasificó como investigación sin riesgo.

## Resultados

En el 2018, se notificaron 1.104 casos de tuberculosis en población privada de la libertad al Sivigila, de los cuales 496 (45 %) pertenecían al régimen especial, 432 (39 %) al subsidiado, 72 (6,5 %) a regímenes de excepción, 48 (4,3 %) a no asegurados y 25 (2,3 %) no registraban información de afiliación al Sistema General de Seguridad Social en Salud (SGSSS).

Al analizar la pertenencia étnica, 57 casos (5,2 %) correspondían a afrocolombianos y 22 (2,0 %) a indígenas, raizales, palenqueros o población romaní. Por grupos edad, el mayor número de casos se concentró en el de 20 a 29 años, con 613 (55,5 %); 328 casos (29,7 %) se dieron en el de 30 a 39 años, en tanto que 153 casos (13,9 %) correspondieron a mayores de 40 años. De los 132 centros carcelarios y penitenciarios, 74 notificaron por lo menos un caso de tuberculosis al Sivigila. De los 1.104 casos de tuberculosis de todo tipo, 997 eran nuevos, cifra que sirvió para calcular la tasa de incidencia en cada establecimiento. En el análisis, se encontró que los establecimientos carcelarios de las ciudades de Honda y Cúcuta contribuyeron con la mayor tasa de incidencia, con un porcentaje de hacinamiento mayor de 50 % (cuadro 2).

**Cuadro 2:** Carga de tuberculosis, tasas de incidencia por 100.000 población privada de la libertad, porcentaje de hacinamiento, capacidad y sobrepoblación para los centros carcelarios y penitenciarios que registraron tuberculosis, Colombia, 2018

Entidad territorial	Centro carcelario y penitenciario	Casos nuevos	Población	Tasa Incidencia *100.000	Capacidad	Sobrepoblación	Hacinamiento %
Antioquia	EPMSC Leticia	2	166	1.204,8	118	48	40,7
	Complejo Penitenciario Medellín-Pedregal	16	2.227	718,5	1.288	939	72,9
	EP Puerto Triunfo	25	1800	1.388,9	1.316	484	36,8
	EPMSC Andes	5	792	631,3	168	624	371,4
	EPMSC Caucasia	3	128	2.343,8	63	65	103,2
	EPMSC La Ceja	2	293	682,6	94	199	211,7
	EPMSC Medellín	62	3.270	1.896,0	1.869	1401	75,0
	EPMSC Puerto Berrío	4	298	1.342,3	150	148	98,7
	EPMSC Santo Domingo	4	272	1.470,6	115	157	136,5
	EPMSC Sonsón	1	213	469,5	75	138	184,0
	EPMSC Yarumal	1	291	343,6	191	100	52,4
Atlántico	EC Barranquilla	11	1.019	1.079,5	454	565	124,4
	EPMSC Barranquilla	27	1715	1.574,3	640	1075	168,0
Bogotá	Complejo Carcelario y Penitenciario Metropolitano de Bogotá	47	8.718	539,1	5.906	2812	47,6
	Establecimiento Carcelario La Modelo	5	5.024	99,5	3.081	1943	63,1
Bolívar	EPMSC Cartagena	8	2.505	319,4	1.386	1119	80,7
Boyacá	EPAMSCAS Cómbita	9	1.705	527,9	1.164	541	46,5
Caldas	Cárcel de Mujeres	1	198	505,1	128	70	54,7
	EPAMS La Dorada	14	1.502	932,1	1.524	-22	-1,4
	EPMSC Anserma	1	181	552,5	128	53	41,4
	EPMSC Manizales	4	1.444	277,0	670	774	115,5
	EPMSC Pensilvania	1	105	952,4	56	49	87,5
Caquetá	EP Las Heliconias de Florencia	13	1.359	956,6	1.388	-29	-2,1
	EPMSC Florencia	2	896	223,2	550	346	62,9
Casanare	EPC Yopal	13	1.219	1.066,4	918	301	32,8
	EPMSC Paz de Ariporo	3	151	1.986,8	120	31	>25,8
Cauca	EPAMSCAS Popayán (ERE)	19	2.614	726,9	2.524	90	3,6
	EPMSC Bolívar-Cauca	1	170	588,2	176	-6	-3,4
	EPMSC Santander de Quilichao	1	493	202,8	230	263	114,3
Cesar	EPAMSCAS Valledupar (ERM)	3	1.122	267,4	256	866	338,3
	EPMSC Valledupar	21	1.383	1.518,4	1.632	-249	-15,3
Chocó	EPMSC Itsmina	1	131	763,4	81	50	61,7
	EPMSC Quibdó	4	619	646,2	286	333	116,4
Córdoba	EPMSC Montería	12	1.731	693,2	840	891	106,1
	EPMSC Tierralta	3	1207	248,6	1.248	-41	-3,3
Cundinamarca	EPMSC Girardot	7	729	960,2	555	174	31,4
	EPMSC La Esperanza Guaduas	42	2.884	1.456,3	2.822	62	2,2
La Guajira	EPMSC Riohacha	2	452	442,5	100	352	352,0
Huila	EPMS - La Plata	1	417	239,8	304	113	37,2
	EPMSC Garzón	1	392	255,1	251	141	56,2
	EPMSC Neiva	16	1.806	885,9	950	856	90,1
	EPMSC Pitalito	1	1.026	97,5	690	336	48,7
Magdalena	EPMSC El Banco	3	179	1.676,0	80	99	123,8
	INPEC Cárcel Santa Marta	26	1.370	1.897,8	312	1.058	339,1
Meta	EPMSC Acacías	6	2.856	210,1	2.376	480	20,2
	EPMSC Villavicencio	9	2.020	445,5	1.003	1.017	101,4
Nariño	EPMSC RM Pasto	3	1.320	227,3	568	752	132,4
	EPMSC Tumaco	2	565	354,0	274	291	106,2
Norte de Santander	Complejo Carcelario y Penitenciario Metropolitano de Cúcuta	70	2.222	3.150,3	1.383	839	60,7
	EPMSC Armenia	4	457	875,3	350	107	30,6
Quindío	EPMSC Calarcá	4	923	433,4	980	-57	-5,8
	EPMSC Pereira (ERE)	31	1.249	2.482,0	676	573	84,8
Risaralda	EPMSC Santa Rosa de Cabal	1	240	416,7	178	62	34,8
	RM Pereira	1	385	259,7	305	80	26,2
	EPMS San Andrés	2	213	939,0	136	77	56,6
San Andrés	EPAMS Girón	4	1.921	208,2	1.622	299	18,4
Santander	EPMS San Gil	3	257	1.167,3	262	-5	-1,9
	EPMSC Barrancabermeja	9	508	1.771,7	200	308	154,0
	EPMSC Bucaramanga (ERE)	34	2.785	1.220,8	1.520	1.265	83,2
	EPMSC Málaga	2	91	2.197,8	60	31	51,7
	EPMSC Socorro	1	492	203,3	318	174	54,7
	RM Bucaramanga	1	2.785	35,9	1.520	1.265	83,2
	EPMSC Sincelejo	6	1.080	555,6	512	568	110,9
Sucre	Complejo Carcelario de Ibagué - Picaleña	49	3.857	1270,4	3.278	579	17,7
Tolima	EPMSC Honda	17	328	5182,9	208	120	57,7
	INPEC Líbano	2	108	1851,9	99	9	9,1
	Complejo Carcelario y Penitenciario de Jamundí	24	2.891	830,2	2.916	-25	-0,9
Valle del Cauca	EPAMS Palmira	26	2.103	1236,3	1.257	846	67,3
	EPMSC Buenaventura	7	611	1145,7	335	276	82,4
	EPMSC Buga	9	1.291	697,1	821	470	57,2
	EPMSC Cali (ERE)	184	5.973	3080,5	2.046	3.927	191,9
	EPMSC Cartago	7	525	1333,3	428	97	22,7
	EPMSC- Sevilla	4	159	2515,7	120	39	32,5
	EPMSC Tulúa	7	1.376	508,7	1.078	298	27,6
Nacional		977	119.172	819,8	80.227	38.945	48,5

PPL: población privada de la libertad; EPMSC: Establecimiento Penitenciario de Mediana Seguridad y Carcelario; EPMS: Establecimiento Penitenciario de Mediana Seguridad; EPMSC - RM: Establecimiento Penitenciario de Mediana Seguridad y Carcelario - Reclusión de Mujeres

Fuente: datos del Sivigila, evento tuberculosis, INS. Población privada de la libertad, INPEC

En negrilla se encuentran los centros carcelarios que no cuentan con un porcentaje de hacinamiento.

**Figura 1:**
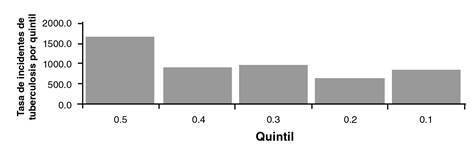
Incidencia de tuberculosis en población privada de la libertad, según porcentaje de hacinamiento en los centros penitenciarios y carcelarios de Colombia, 2018

La incidencia de tuberculosis en la población privada de la libertad en centros penitenciarios y carcelarios agrupada por quintiles según el porcentaje de hacinamiento, evidenció que en el quintil 5 se agruparon los de mayor hacinamiento y, en el quintil 1, los de menor hacinamiento; esto evidenció que la incidencia y el peso porcentual son mayores en los centros con más hacinamiento que en aquellos con menos (figura 1).

Fuente: datos del Sivigila, evento tuberculosis, INS. Población privada de la libertad, INPEC

La diferencia en la incidencia de tuberculosis en la población privada de la libertad entre el quintil 5 y el 1, fue de 798 casos por cada 100.000 personas, lo que indica que el hacinamiento carcelario es una condición social que favorece la infección por *Mycobacterium tuberculosis*. Asimismo, al calcular la desigualdad relativa entre los dos grupos, se encontró que la incidencia de la enfermedad en la población con mayor hacinamiento fue 1,92 veces o 92 % más alta que la incidencia en el grupo con menor hacinamiento (cuadro 3).

**Cuadro 3:** Tasa de incidencia de tuberculosis por quintil y desigualdad absoluta y relativa de los centros carcelarios y penitenciarios que registraron tuberculosis, Colombia, 2018

Tasa de incidencia de tuberculosis por quintil	
Q5 - Grupo con mayor porcentaje de hacinamiento 1665,61	Q1 - Grupo con menor porcentaje de hacinamiento 866,8
Desigualdad absoluta IC95% 798,8 (761,32 - 838,07)	
Desigualdad relativa IC95% 1,92 (1,83 - 2,01)	

Fuente: datos del Sivigila, evento tuberculosis, INS. Población privada de la libertad, INPEC

**Figura 2:**
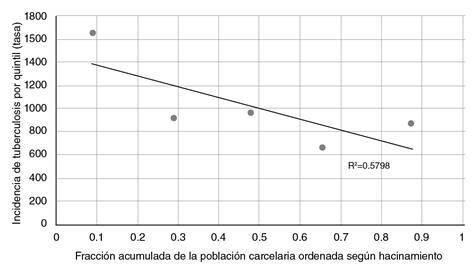
Índice de desigualdad de la pendiente: desigualdad absoluta en la incidencia de tuberculosis en población privada de la libertad, según porcentaje de hacinamiento en Colombia, 2018

El índice de desigualdad de la pendiente es una medida que representa la diferencia absoluta entre los valores de un indicador en salud, en este caso, la incidencia de tuberculosis entre la población privada de la libertad con un nivel más alto de hacinamiento y aquellos con uno más bajo, mediante el uso de un modelo de regresión lineal simple. En este estudio, este índice fue de -724,9, es decir, hubo un exceso de 724 casos de la enfermedad por cada 100.000 en la población con mayor concentración de hacinamiento, comparada con la de menor hacinamiento, considerando la información de todos los grupos y su peso poblacional (figura 2).

Fuente: datos del Sivigila, evento tuberculosis, INS. Población privada de la libertad, INPEC

**Figura 3:**
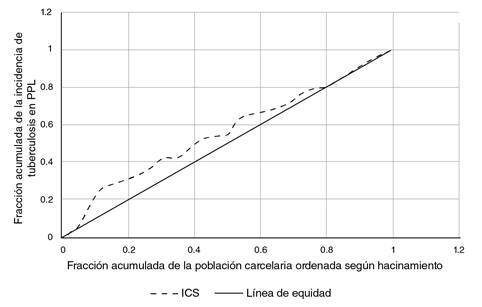
Desigualdad relativa en la incidencia de tuberculosis según porcentaje de hacinamiento, representada con la curva de concentración de población privada de la libertad, 2018

Por otra parte, el índice de concentración en salud, que refleja el gradiente de desigualdad absoluta entre el grupo más aventajado (menor hacinamiento) y el más desaventajado (mayor hacinamiento), fue de -0,121, lo que indica que la incidencia de la enfermedad se concentró en el grupo con mayor porcentaje de hacinamiento. En la figura 3, la línea punteada representa el gradiente de desigualdad tomando como referencia la línea gris, correspondiente a la línea de equidad en la que no existiría desigualdad entre los grupos.

Fuente: datos del Sivigila, evento tuberculosis, INS. Población privada de la libertad, INPEC

## Discusión

El objetivo de este estudio fue estimar las condiciones de hacinamiento carcelario como inequidad social en salud en la población privada de la libertad con tuberculosis, mediante medidas de desigualdad simples, como las brechas absolutas y relativas, y medidas complejas, como el índice de la pendiente y el índice de concentración. Los resultados permitieron determinar que, en Colombia, existen inequidades sociales con impacto en la salud de la población privada de la libertad, lo que refleja una grave problemática social, ya que más del 94 % de los establecimientos carcelarios y penitenciarios registraron una sobrepoblación de 39.773 personas en el 2018, es decir, un hacinamiento del 48 % ^19^, condiciones estas que favorecen el aumento del riesgo de infección por *M. tuberculosis*^8^^,^^20^^,^^21^.

Las brechas absoluta y relativa entre el grupo con mayor hacinamiento y el de menor hacinamiento, permitieron establecer que existen diferencias en la incidencia de la tuberculosis (798 casos por cada 100.000 población privada de la libertad). Esta situación también refleja la desigualdad relativa, pues la incidencia de la enfermedad en la población privada de la libertad con mayor hacinamiento fue casi dos veces mayor que la del grupo con menor sobrepoblación. En este sentido, Fazel, *et al*., han reportado tasas elevadas de tuberculosis en países pobres, en donde los centros penitenciarios actúan como reservorios de la infección con riesgo de transmisión hacia las comunidades vecinas ^22^^,^^23^.

Otra medida importante es el índice de concentración en salud, que fue de -0,121 en este estudio, lo cual indica que la incidencia de tuberculosis se concentró en el grupo con mayor porcentaje de hacinamiento y sobrepoblación. En cuanto a la enfermedad en los centros carcelarios de Colombia, este índice permite evidenciar la desigualdad de su distribución en la población privada de la libertad debida al hacinamiento, y ratifica cómo las condiciones sociales en las cárceles pueden propiciar la propagación de enfermedades debido a la escasa ventilación, la mala nutrición, y la atención médica inapropiada o inaccesible. Esta situación aumenta la probabilidad de contraer tuberculosis en una prisión donde el hacinamiento sea mayor ^11^^,^^13^^,^^14^.

El hacinamiento es un problema de la mayoría de los centros penitenciarios del país y constituye un factor de riesgo para una enfermedad como la tuberculosis, la cual se considera un grave problema de salud pública, el cual puede tornarse incluso más crítico cuando hay brechas relacionadas con el diagnóstico oportuno, el seguimiento de casos y contactos, el suministro del tratamiento y la ausencia de lugares apropiados para el aislamiento. La OMS resalta que las prisiones representan un reservorio para la transmisión de tuberculosis ^10^^,^^23^ y, conjuntamente con la ONU, ha instado a los países a establecer estrategias que permitan disminuir la brecha de las desigualdades en salud de esta población en evidente situación de vulnerabilidad debido a su mayor exposición.

Los estudios que abordan la problemática social del hacinamiento desde la perspectiva de las inequidades en salud y su medición, aún son escasos. Algunos se han enfocado en la relación entre la tuberculosis y el hacinamiento como factor de riesgo en la población privada de la libertad y, en ellos, se ha concluido que el aumento del contacto físico, la falta de ventilación, así como el poco tiempo al aire libre, favorecen la propagación de las enfermedades infecciosas. En Brasil ^14^, por ejemplo, se estableció que los síntomas de la tuberculosis pulmonar son 35 a 39 veces más frecuentes en las cárceles sobrepobladas que en la población general. Asimismo, en el estudio de Hussein, *et al*., en Pakistán, se encontró que el riesgo de una infección tuberculosa latente era casi tres veces mayor en los reclusos alojados en áreas de menos de 18 metros ^22^. Algunos estudios en Colombia se han enfocado en estrategias comunitarias de prevención en salud y en los factores de riesgo relacionados con la tuberculosis pulmonar en la cárcel de Villahermosa de Cali ^24^ y con el hacinamiento en la cárcel La Cuarenta de Pereira ^25^. En los dos estudios, se concluyó que la prevalencia de todas las infecciones de transmisión aérea aumentaba en ambientes superpoblados o con ventilación inadecuada ^8^. Otros estudios sobre tuberculosis y población privada de la libertad en el país, coinciden en cuanto a la alta prevalencia de esta enfermedad en instituciones carcelarias comparada con la de la población general ^26^^,^^27^^,^^28^, situación en la que tiene un papel significativo el hacinamiento o sobrepoblación.

El presente estudio tuvo algunas limitaciones que deben considerarse. Por un lado, a pesar de contar con el reporte general de casos de tuberculosis en la población privada de la libertad notificados a nivel nominal al Sivigila, no todos los centros carcelarios los reportaron, lo que se explicaría por la ausencia de búsquedas activas en la vigilancia epidemiológica y plantea la posibilidad de subregistros. Por otra parte, la información que tiene el INPEC carece de datos desagregados a nivel de cada centro carcelario por grupos de edad, afiliación a entidades administradoras de planes de beneficios, datos sobre servicios de salud, suministro y seguimiento de tratamiento.

En este sentido, en futuros estudios deben considerarse otras variables de interés en la relación entre la tuberculosis en la población carcelaria y las condiciones de hacinamiento. Por ejemplo, debe contemplarse una probable mayor incidencia de la enfermedad en las ciudades de origen de los reclusos, pues ello incidiría en su presencia en los centros penitenciarios, así como las características o condiciones nutricionales de esta población. Si se incorporan estas y otras variables, se obtendrían diseños metodológicos multivariados que, sin duda, contribuirían a la comprensión integral de la problemática central de este estudio.

En conclusión, el presente estudio tuvo como objetivo estimar las inequidades sociales de la población privada de la libertad que padece tuberculosis y que vive bajo condiciones de hacinamiento en centros carcelarios del país. Estas desigualdades son injustas y evitables, ya que hay medidas de control y prevención de la enfermedad, y pueden adoptarse políticas y programas que reduzcan el hacinamiento y mejoren las condiciones de vida en las cárceles ^25^. Es importante ejecutar estrategias multisectoriales para mejorar la salud de la población privada de la libertad y reducir estas brechas de forma significativa. Asimismo, es indispensable continuar haciendo estudios que determinen la relación de las condiciones de salud de la población privada de la libertad en situación de hacinamiento y otras circunstancias difíciles de su entorno desde la perspectiva de las desigualdades sociales.
